# Influence of “cryoconcentration” on the composition of bacterial communities in semi-enclosed shallow water lakes

**DOI:** 10.3389/fmicb.2025.1623773

**Published:** 2025-07-23

**Authors:** Xu Bingxian, Shi Yujiao, Li Wenbao, Gao Hengshuai

**Affiliations:** ^1^State Key Laboratory of Water Engineering Ecology and Environment in Arid Area, Inner Mongolia Agricultural University, Hohhot, China; ^2^Inner Mongolia Wild Scientific Observatory on the Ecological Environment of the Dali-nor Lake, Chifeng, China; ^3^Autonomous Region Collaborative Innovation Center for Integrated Management of Water Resources and Water Environment in the Inner Mongolia Reaches of the Yellow River, Hohhot, China

**Keywords:** freezing process, bacterial community structure, ice-water interface, co-occurrence network, Assembly process

## Abstract

Bacteria serve as vital indicators of the lake ecosystem health. Although substantial progress has been made in investigating the structural features of lake bacterial communities, limited attention has been paid to the dynamic assembly processes and driving factors affecting bacterial communities in ice and water environments during the freeze-up period. In this study, we investigated aggregation and compositional changes in bacterial communities in the internal ice-covered state of Lake Hulun. We examined the effects of cryoconcentration under low-temperature conditions on community assembly and systematically analyzed the physicochemical parameters as well as α- and β-diversity of bacterial communities in bottom ice (BI) and surface water (SW) media. Bacterial diversity was significantly higher in SW than in BI. Among the dominant taxa, eight phyla were shared between both environments. Firmicutes and Patescibacteria were dominant in the BI, whereas Gemmatimonadota and Bdellovibrionota were dominant in the SW. Nutrient transport driven by cryoconcentration emerged as a key factor influencing bacterial community assembly. Specifically, total nitrogen and salinity regulated the balance between stochastic and deterministic processes in BI and SW, respectively. Overall, the distinct environmental conditions of BI and SW weakened the diffusion capacity of bacterial communities, resulting in diffusion-limited and drift-dominated assembly processes. These findings offer new insights into the mechanisms underlying bacterial interactions and community assembly in ice-covered lake habitats and provide a scientific foundation for the management and preservation of lake ecosystems under ice-covered conditions.

## Introduction

Lakes are a vital component of regional aquatic ecosystems, characterized by frequent exchanges of matter and energy with the surrounding environment, and are often considered as early warning indicators of environmental change (Williamson et al., 2008; Adrian et al., 2009; Hodson et al., 2017). Globally, approximately 50 million lakes have undergone seasonal ice formation, a phenomenon that plays a defining role in shaping the landscapes of northern China. In these regions, ice cover can persist for 5–7 months, forming a physical barrier that restricts the exchange of heat, gases, and nutrients between the atmosphere and lake water. During the process, the “sewage” of “freezing and concentration” drives the accumulation of nitrogen, phosphorus, and other nutrients into the water column, leading to pronounced shifts in the lake’s environmental conditions (Bramburger et al., 2023). Bacterial communities are crucial for maintaining ecosystem stability and are highly responsive to environmental change (Williamson et al., 2008; Guo et al., 2018). Subject to multiple environmental influences, they can adjust in response to perturbation. Notably, in ice-covered lakes, bacterial growth, metabolic activity, and organic matter accumulation continue under reduced light conditions, thereby enhancing their role in the subglacial food web and giving rise to a unique ecological system (Zhang et al., 2019). Although numerous studies have examined the transport and transformation of nutrients such as nitrogen and phosphorus in open-water systems, limited attention has been paid to the composition and metabolic potential of bacterial communities in ice-water environments affected by natural cryoconcentration processes. This knowledge gap restricts our understanding of how bacterial communities respond to habitat changes during ice-covered periods.

Currently, high-throughput sequencing has become a widely used tool to assess bacterial community diversity in lakes. Analyzing how bacterial communities respond to both biotic and abiotic factors has become a central focus in aquatic ecology and environmental research (Yang et al., 2022). Understanding the structural characteristics of bacterial communities in distinct lake media and elucidating the underlying community assembly mechanisms are essential for maintaining the ecological integrity of freshwater systems. In general, factors that can affect bacterial community structure involve multiple ecological processes, including selection, drift, speciation, and dispersal, which can result from both deterministic and stochastic mechanisms (Hodson et al., 2017). Deterministic processes refer to environmental selection and biotic interactions (including competition, predation, and symbiosis), whereas stochastic processes include random events such as birth, death, dispersal, and extinction (Hanson et al., 2012; Hodson et al., 2017; Liu et al., 2018). Recent studies have shown that deterministic processes often dominate the shaping of bacterioplankton communities in shallow lakes (Jiao et al., 2020), whereas spatial variability in community composition can also be significantly influenced by stochastic forces (Bai et al., 2020; Xian et al., 2024). As key biogenic elements, nitrogen and phosphorus are closely associated with bacterial abundance and diversity, particularly in coastal zones (Wang et al., 2020). Seasonal variations in hydrology, such as dry and wet periods, also significantly affect both α-diversity and spatial heterogeneity in bacterial communities (Kim et al., 2019). Environmental heterogeneity and nutrient preferences across different habitats are important drivers of shifts in community composition and spatial distribution patterns (Jansen et al., 2021). However, most existing studies have focused on the assembly patterns of bacterial taxa in open-water aquatic ecosystems. Far less attention has been paid to bacterial communities in ice-water environments of ice-covered lakes, particularly in terms of how cryoconcentration and elemental transport influence community assembly at the ice-water interface.

Lakes in Inner Mongolia represent vital freshwater reserves in the ecologically fragile regions of northern China and play a crucial role in maintaining ecological balance. Lake Hulun experiences pronounced seasonal variability as a typical high-latitude cold region lake. It remains frozen for 5–6 months each year, during which cryoconcentration under the ice cap facilitates nutrient migration and exacerbates eutrophication of the underlying water column. Although previous studies have examined the microbial communities in Lake Hulun, little attention has been paid to microbial dynamics within the ice–water habitat, particularly under the influence of prolonged ice cover. In response, this study focused on Lake Hulun in northeastern Inner Mongolia. Using stratified sampling of lake ice and under-ice water, bacterial communities were characterized via Illumina MiSeq high-throughput sequencing, along with measurement of key physicochemical parameters, including pH, dissolved oxygen (DO), salinity (SAL), total nitrogen (TN), and total phosphorus (TP). The specific objectives of this study were to: (1) characterize the composition and structural differences in bacterial communities between ice and water media during the ice-covered period; (2) identify the environmental drivers, particularly nutrient gradients and ice–water interfaces, which could influence bacterial community differentiation across habitats; and (3) evaluate how the community assembly mechanisms and biotic interactions could vary between nutrient-poor (ice) and eutrophic (water) environments and how these processes could affect the ecological homeostasis of the lake. These insights provide a theoretical foundation for the ecological assessment and integrated management of lakes in cold and arid regions.

## Materials and methods

### Regional overview and sample collection

Lake Hulun is located in Hulunbuir City, between Xin Barag Right Banner, Xin Barag Left Banner, and Zhalainuoer District of Manzhouli City (48°33′–49°20′N, 116°58′–117°48′E). It is the largest lake in Inner Mongolia and the fifth largest freshwater lake in China (Cai et al., 2016). The Mongolian Plateau lies to the west and south of the basin, whereas the Xing’an ling Mountains border it to the east. The lake has an irregular and elongated shape, with a surface area of approximately 2,080 km^2^. It extends approximately 93 km in length, with a maximum width of 41 km and an average width of 32 km. Its shoreline perimeter measures approximately 480 km. Based on monitoring data from 2014 to 2019, the average water depth is approximately 5.33 m, whereas the maximum depth can reach up to 8 m during periods of high water levels. The current estimated water storage capacity is approximately 13.85 billion m^3^.

**FIGURE 1:**
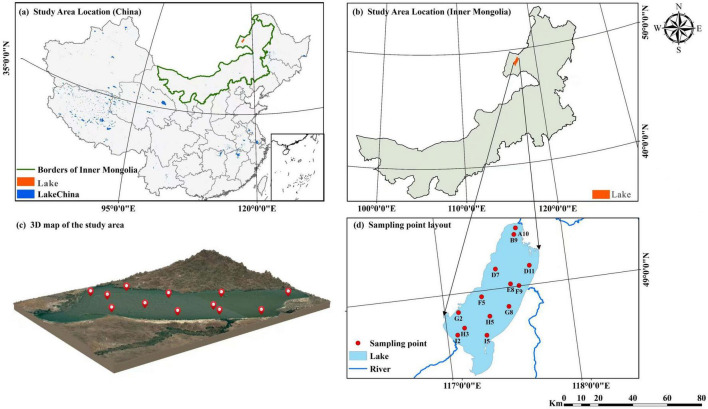
**(a)** Location of the study area in China; **(b)** geographic location of Lake Hulun in Inner Mongolia, China; **(c)** 3D topographic map of the study area; **(d)** spatial distribution of sampling sites in Lake Hulun.

Lake Hulun is a typical lake in a cold and arid region, characterized by an annual ice cover of 7 months and ice thickness exceeding 1 m, with minimal exposure to anthropogenic influences. This provides a natural context for investigating the structural characteristics of bacterial communities and their responses to habitat transitions between ice and water media under prolonged ice cover conditions. Considering the integrated icing processes, a total of 13 sampling sites (Figure 1) were established. Sampling was conducted in January 2023. At each site, two types of samples were collected: bottom ice (BI) and surface water (SW). BI samples were obtained by drilling boreholes through ice using an ice auger and collecting approximately 5 cm of ice from the bottom of the ice layer. SW samples were collected from under-ice water, approximately 5–10 cm below the ice-water interface, using a water sampler. A total of 26 valid samples (13 BI and 13 SW) were collected. These samples were used for physicochemical analyses and bacterial community profiling. For microbial analysis, water and melted ice samples were filtered through 0.22 μm sterile microfiltration membranes to retain the bacterial cells for subsequent DNA extraction.

### DNA extraction, sequencing, and environmental characterization

DNA was extracted from each sample following the method described by Liang et al. (2012). The quality of the extracted DNA was assessed by 1% agarose gel electrophoresis. The V3–V4 hypervariable region of the bacterial 16S rRNA gene was amplified using the primer pair 335F (5′-ACTCCTACGGGAGGCAGCAG-3′) and 806R (5′-GGACTACHVGGTWTCTAAT-3′) (Walters et al., 2016). PCR amplification was conducted under the following thermal cycling conditions: initial denaturation at 95°C for 3 min, followed by 27 cycles of denaturation at 95°C for 30 s, annealing at 55°C for 30 s, and extension at 72°C for 30 s, and a final extension at 72°C for 10 min. The amplified products were stored at 4°C until further analysis. For detailed amplification conditions and reagent composition, please refer to the protocol outlined in the cited references (Magoc and Salzberg, 2011; Edgar, 2013; Zhou et al., 2021). Sequencing data processing methods are detailed in a study by Shi et al. (2024).

Physicochemical parameters, including pH, dissolved oxygen (DO), salinity (SAL), total nitrogen (TN), and total phosphorus (TP), were determined in accordance with the fourth edition of the Methods for the Analysis of Water and Wastewater Monitoring.

### Data processing

The alpha diversity metrics, including ACE and Simpson indices, were calculated using Mothur software (v1.30.2). Principal coordinate analysis (PCoA), bacterial community composition profiling, and comparative statistical analyses were conducted in R (v3.3.1) using vegan, ggplot2, stats, Hmisc (v5.0.1), and minpack.lm (v1.2.3) packages. LEfSe was employed to identify differentially abundant taxa across taxonomic levels, and linear discriminant analysis (LDA) was applied to estimate the effect size of biomarkers distinguishing different groups. Redundancy analysis (RDA) was used to examine the relationships between environmental factors and bacterial phyla, using the “vegan” package (version 2.4-3) in R. Based on Spearman’s correlation coefficients among bacterial taxa, an ice-water bacterial co-occurrence network was constructed. Highly correlated and taxonomically diverse species were selected to investigate molecular ecological network structures across different media. The community composition was analyzed using the R packages Hmisc (v5.0.1) and minpack.lm (v1.2.3), and the key topological properties for network visualization were calculated in Cytoscape (v3.10.2). Based on the intra-module (Zi) and inter-module (Pi) connectivity, the nodes were categorized into module hubs (Zi > 2.5, Pi < 0.62), connectors (Zi < 2.5, Pi > 0.62), network hubs (Zi > 2.5, Pi > 0.62), and peripherals (Zi < 2.5, Pi < 0.62), with the first three considered key taxa. To assess community assembly mechanisms, a neutral community model was constructed using operational taxonomic unit (OTU) abundance tables and phylogenetic trees. This model assumed that stochastic processes, including birth, death, and migration, could drive community structure and fit the observed abundance-frequency distributions to a beta distribution derived from neutral theory. The relative abundance-frequency distributions of different taxa, as observed in microbiological studies, were fitted to a beta distribution derived from the neutral theory. The comparative significance of various ecological processes in shaping biological community assembly was further assessed using iCAMP (1.3.4) (Ning et al., 2020). In this method, species were initially grouped into distinct bins based on a phylogenetic tree. The species with the highest relative abundance in each bin was designated as the central taxon. Other species were assigned to the same bin if their phylogenetic distance to the central species was less than the defined threshold (ds = 0.2). Otherwise, a new bin was created. For each bin, two indices were calculated based on a null model: βNTI and RCbray. The ecological processes were inferred as follows: βNTI values less than −1.96 indicated homogeneous selection (HoS), while values greater than + 1.96 represented heterogeneous selection (HeS). When |βNTI| ≤ 1.96, the classification relied on RCbray values, where RCbray < −0.95 denotes homogeneous dispersal (HD), RCbray > +0.95 indicates dispersal limitation (DL), and |RCbray| ≤ 0.95 was interpreted as drift (DR). These analyses were repeated for all bins, and the relative abundance of each bin was used to weigh and summarize the contribution of each ecological process at the community level.

## Results and analyses

### Differences in physical and chemical indicators between BI and SW

**FIGURE 2:**
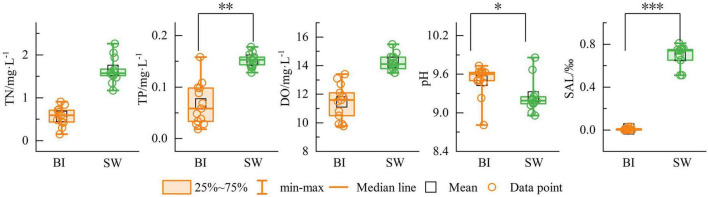
Contents of environmental indicators in BI and SW samples. *p ≤ 0.05, **p ≤ 0.01, ***p ≤ 0.001.

During the freezing process, the cryoconcentration effect resulted in marked differences in physicochemical parameters between the BI and SW samples (Figure 2). The mean TN concentrations were 0.57 mg/L in BI and 1.64 mg/L in SW. The mean TP concentrations were 0.07 mg/L and 0.15 mg/L, respectively. DO levels averaged 11.46 mg/L in BI and 14.22 mg/L in SW. The mean SAL levels were 0.008‰ in BI and 0.69‰ in SW. Overall, the values of TN, TP, DO, and SAL in SW were significantly higher than those in BI. In contrast, pH was slightly higher in BI (9.50) than in SW (9.25), although the difference was not statistically significant (Figure 2).

### Comparison of bacterial community diversity between BI and SW

**FIGURE 3:**
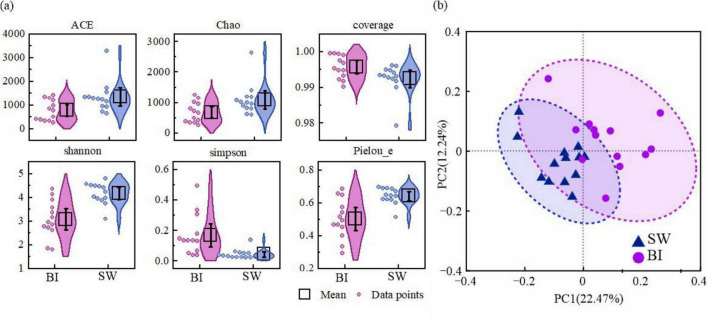
**(a)** α-Diversity indices of bacterial communities in BI and SW; **(b)** principal coordinate analysis (PCoA) based on Bray–Curtis dissimilarity showing intergroup differences in bacterial communities, with ellipses indicating 95% confidence intervals.

The *alpha* diversity of bacterial communities in the BI and SW samples was assessed using a series of indices, including richness estimators (ACE and Chao1), diversity indices (Shannon and Simpson), coverage, and Pielou’s evenness (Pielou_e). These indices revealed that the bacterial diversity and richness varied significantly between the two environments (Figure 3a). In addition, PCoA was performed to analyze bacterial community structures. The results demonstrated distinct community composition patterns associated with different BI and SW media (Figure 3b).

The ACE and Chao1 richness indices were significantly higher in the SW samples (mean: 1345.53 and 1096.43, respectively) than in the BI samples (mean: 782.10 and 680.31, respectively), indicating greater bacterial richness in the water column. In contrast, the Shannon diversity index was significantly higher in SW (4.16) than in BI (3.08, *p* < 0.001), whereas the index was lower in SW (0.04) than in BI (0.17, *p* < 0.01), suggesting a markedly higher diversity of bacterial communities in SW (Figure 3a). The sequencing coverage was above 99% in both environments (99.6% in BI and 99.2% in SW), indicating that the sequencing depth was sufficient to capture the majority of microbial diversity (Figure 3a). The Pielou_e index was also slightly higher in SW (mean: 0.64) than in BI (mean: 0.50), implying a more evenly distributed bacterial community in the water environment. Furthermore, PCoA revealed a clear separation between BI and SW samples, confirming significant compositional differences in bacterial communities between the two BI–SW media (Figure 3b).

### Differences in community structure and indicator species between BI and SW

**FIGURE 4:**
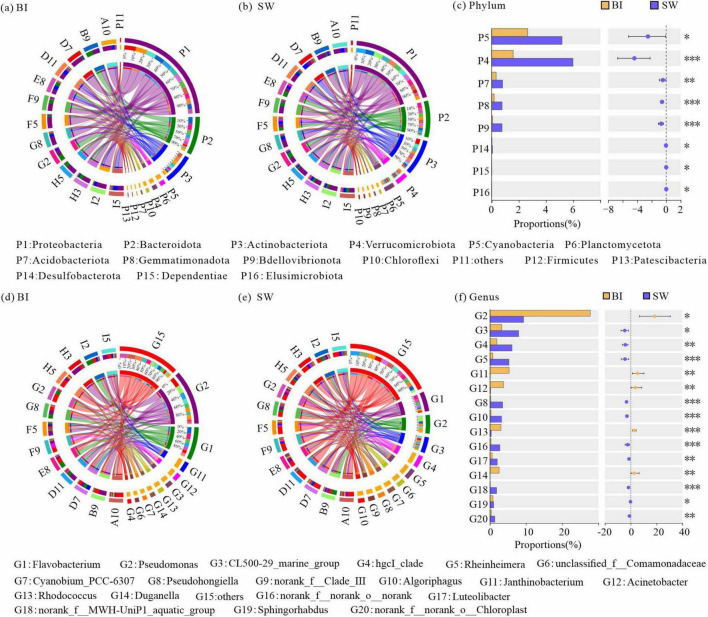
**(a,b)** Relative abundances of bacterial communities at the phylum level in BI **(a)** and SW **(b)** samples; **(c)** significance testing of intergroup differences in bacterial phyla between BI and SW samples; **(d,e)** relative abundances of bacterial communities at the genus level in BI **(d)** and SW **(e)** samples; **(f)** significance testing of intergroup differences in bacterial genera between BI and SW samples. Only dominant phyla and genera are displayed; taxa with relative abundances < 1% are grouped into the category “others.” For **(a,b)** and **(d,e)**, the left half-circle indicates the taxonomic composition at each sampling points, with different colors representing different sampling points and taxa in outer and inner rings, respectively; the right half-circle indicates the overall taxonomic composition in all BI or SW samples as well as the distribution of each taxon across different sampling points, with different colors representing different taxa and sampling points in outer and inner rings, respectively. *p ≤ 0.05, **p ≤ 0.01, ***p ≤ 0.001.

A total of 3,558 OTUs were identified across all BI and SW samples, encompassing 66 bacterial phyla. These OTUs were further classified into 164 classes, 372 orders, 598 families, 1,020 genera, and 1,765 species. Among them, Proteobacteria, Bacteroidota, Actinobacteriota, Cyanobacteria, Verrucomicrobiota, Planctomycetota, Acidobacteria, and Chloroflexi exhibited the highest relative abundances and were considered the dominant bacterial phyla, defined as those with a mean relative abundance greater than 1% in both BI and SW samples (Figures 4a, b).

Furthermore, the Wilcoxon rank-sum tests demonstrated that the relative abundances of five dominant bacterial phyla and three rare phyla were significantly higher in SW than in BI (Figure 4c). In addition, several dominant genera, including *Pseudomonas*, and rare genera such as *Luteolibacter* also exhibited significant differences in abundance in both BI and SW samples (Figures 4d, e). These results suggested that both dominant and rare bacterial genera presented significant differences in their relative abundances in BI and SW (Figure 4f).

**FIGURE 5:**
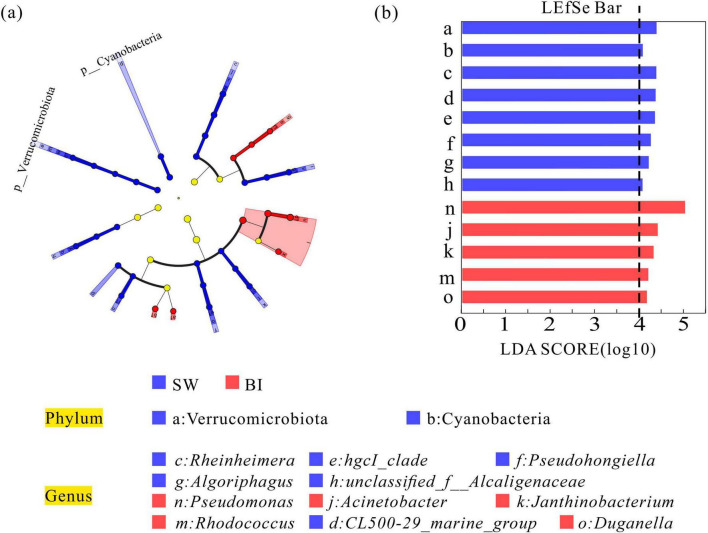
**(a)** Linear discriminant analysis (LDA) illustrating differences in bacterial community composition between BI and SW samples. **(b)** LDA scores (threshold = 4). Higher LDA scores indicate a greater contribution of specific taxa to observed group differences.

To further elucidate the effects of distinct survival environments, the bacterial communities in BI and SW samples were analyzed to identify biomarker taxa. The analysis revealed two biomarker phyla and six biomarker genera that were differentiated between the two media. At the phylum level, Cyanobacteria and Verrucomicrobiota exhibited significantly higher relative abundances in SW samples. At the genus level, *Rheinheimera* and *Pseudohongiella* were among the dominant taxa in SW. In contrast, five genera, including *Pseudomonas*, *Acinetobacter*, *Janthinobacterium*, *Rhodococcus*, and *Duganella*, were significantly more abundant in BI samples (Figures 5a, b).

Overall, the bacterial community structure differed markedly between BI and SW samples. The identification of eight dominant phyla and several habitat-specific genera highlighted the significant role of ice formation and environmental conditions in shaping microbial community composition across ice and water habitats.

### Characteristics of ecological network in BI and SW samples

**FIGURE 6:**
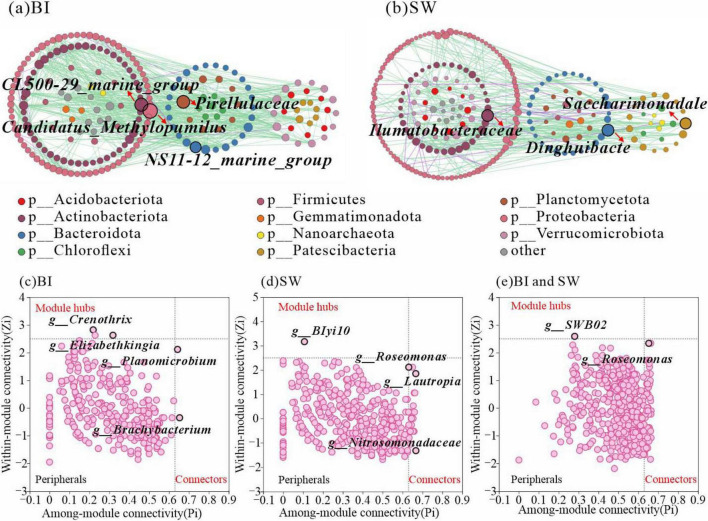
**(a,b)** Co-occurrence networks of bacterial communities in BI **(a)** and SW **(b)**. Each node represents a bacterial genus that is colored according to phylum affiliation. The node size is proportional to the node degree. Edges represent significant interactions between genera (*p* < 0.05), with green indicating positive correlations and purple indicating negative correlations. **(c–e)** Zi-Pi scatter plots showing intra-module connectivity (Zi) versus inter-module connectivity (Pi) of key taxa in the network. Highlighted taxa include *Crenothrix*, *Elizabethkingia*, *BIyi10*, *Roseomonas*, *Lautropia*, *Nitrosomonadaceae*, *SWB02* (Proteobacteria), *Planomicrobium* (Firmicutes), and *Brachybacterium* (Actinobacteriota).

Ecological network analysis revealed notable structural differences between BI and SW samples. The BI network comprised 221 nodes and 718 edges with a network density of 0.015, whereas the SW network contained 243 nodes and 387 edges with a lower density of 0.007. These results indicated that the bacterial community in BI exhibited a higher overall connectivity. However, the bacterial interactions in both habitats were primarily driven by synergistic relationships. Analysis of node degree and topological roles identified *Candidatus_Methylopumilus* (Proteobacteria) and *CL500-29_marine_group* (Actinobacteriota) as key taxa contributing to the structural stability of the BI network (Figure 6a). In contrast, *Dinghuibacter* (Bacteroidota) emerged as a central genus for maintaining the integrity of the SW ecological network (Figure 6b).

The Zi-Pi analysis indicated that Crenothrix, Elizabethkingia, Planomicrobium, and Brachybacterium acted as the inter-module connectors in the BI samples, while *BIyi10*, *Roseomonas*, and *Lautropia* played similar roles in the SW samples (Figures 6c, d). A total of 27 key genera were identified as the major contributors to the stability of the SW ecological network, with 10 belonging to Proteobacteria and 5 belonging to Verrucomicrobiota. In comparison, only six key genera were identified in the BI network and were assigned to Firmicutes, Actinobacteriota, and Proteobacteria. Notably, 65 key genera were located at the BI-SW interface, spanning 12 bacterial phyla. Among them, Proteobacteria, Firmicutes, and Actinobacteriota emerged as the core phyla contributing to network stability at the interface. Additionally, genera such as *SWB02* and *Roseomonas* were identified as key genera linking the BI and SW microbial communities (Figure 6e).

### Mechanism of bacterial community assembly in the BI and SW samples

**FIGURE 7:**
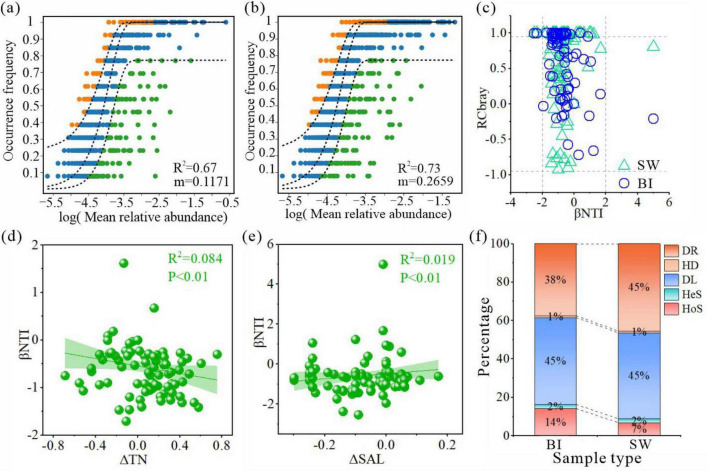
**(a,b)** Neutral community model results for bacterial assemblages in BI **(a)** and SW **(b)**. The solid blue line indicates the model fit, and the dashed line represents the 95% confidence interval. Orange and green points denote the taxa frequencies above and below the model expectations, respectively. **(c)** Scatter plot of Raup–Crick (RC) and β-nearest taxon index (βNTI) values for BI and SW samples. **(d,e)** Relationships between βNTI values of bacterial communities and significant environmental factors in BI **(d)** and SW **(e)** samples. Only the environmental factors with statistically significant correlations are shown. **(f)** Relative contributions (%) of five ecological processes: homogeneous selection (HoS), heterogeneous selection (HeS), homogeneous dispersal (HD), dispersal limitation (DL), and drift (DR).

The analysis demonstrated that both BI and SW samples exhibited strong model fits (R^2^ > 0.5), indicating that stochastic processes played a dominant role in shaping the bacterial community structure in both environments (Figures 7a, b). The influence of stochasticity was greater in SW than BI, whereas the diffusion of species appeared to be more restricted in BI. The relative significance of ecological processes in bacterial community assembly was further quantified using iCAMP analysis, which integrates the βNTI and RCbray indices to jointly identify distinct assembly mechanisms (Figure 7c). The results indicated that stochastic processes played a dominant role in shaping bacterial communities in both ice and water environments, with their influence notably higher in water bodies. Further analysis revealed that stochastic processes were primarily driven by DL and DR. Specifically, the DL had a stronger impact on the BI-associated bacterial community, accounting for 45.2% of community variation, whereas the DR exerted a greater influence on SW, contributing 45.4%. Among the deterministic processes, HoS affected bacterial communities in both media, with a more pronounced effect observed in the ice environment (14.1%) (Figure 7f).

## Discussion

### Effects of “cryoconcentration” on bacterial communities and ecological networks

Lake Hulun experiences ice cover for approximately 5–7 months each year, during which the cryoconcentration effect leads to substantial variations in environmental parameters between the BI and SW samples. These changes in physicochemical factors, such as SAL, pH, DO, TP, and TN, can trigger adaptive responses in bacterial communities. In response to these altered environmental conditions, the bacterial communities exhibited specific structural and compositional adjustments, resulting in distinct community profiles between ice and water habitats (Figure 4).

Specifically, the icing process facilitated the migration of nutrients, such as nitrogen and phosphorus, from ice to water, making the SW environment more conducive to bacterial growth and reproduction. In contrast, nutrient-poor conditions in BI limited the phylogenetic diversity of bacterial taxa (Sang et al., 2021). This was reflected by the higher Chao1 and Shannon diversity indices observed in SW than in BI (Figure 3a). Proteobacteria and Bacteroidota, recognized as the key decomposers in lake ecosystem food webs (Dang and Lovell, 2016), remained the dominant bacterial phyla in both BI and SW samples (Figures 4a, b). The mean pH in both layers was above 9, creating weakly alkaline conditions favorable for Actinobacteriota. Despite similar relative abundances in BI and SW samples, Actinobacteriota emerged as a keystone taxon in stabilizing the BI-SW interface network. This could be due to its metabolic versatility and capacity to mediate the cycling of essential biogenic elements (carbon, nitrogen, and phosphorus) through organic matter decomposition, which plays an indispensable role in the stabilization of ecological networks (Shu et al., 2018). In the SW samples, Verrucomicrobiota played a crucial bridging role in the ecological network and showed a significantly higher abundance, likely due to its involvement in carbon and sulfur cycling (Lin et al., 2004; Mootapally et al., 2025) and its ability to efficiently utilize migrated nutrients. The succession of key taxa in both BI and SW environments is often strongly influenced by habitat heterogeneity. Notably, previous studies have reported that the relative abundance of Chloroflexi can exceed 15% in environments such as alkaline hot springs and alpine tundra, suggesting that this phylum may perform specialized ecological functions under extreme conditions (Klatt et al., 2011; Klatt et al., 2013). This may explain the role of Chloroflexi as a hub taxon linking different modules within the BI network. At the genus level, dominant taxa in BI samples, such as *Pseudomonas* and *Acinetobacter*, are denitrifying (Xia et al., 2020) and characterized by high cold tolerance and adaptability to low-temperature environments. In contrast, genera such as *Rheinheimera* and *Pseudohongiella* are prevalent in SW samples and appear to benefit from higher levels of dissolved oxygen and nutrient availability. These taxonomic shifts may align with elemental transport dynamics under ice cover and reflect habitat-specific bacterial adaptations to distinct environmental conditions.

From the perspective of bacterial co-occurrence networks, the interaction patterns among potentially functional taxa can also play an important role in shaping community structure. Although the ecological network in BI samples exhibited higher complexity, bacterial diversity was significantly greater in the SW samples. This observation suggests that higher microbial diversity does not necessarily correlate with more complex network structures (Tu et al., 2020), potentially owing to differences in energy dynamics (Wright, 1983). The higher primary productivity and species diversity in SW offer different abundant energy and nutrient sources, allowing bacteria to rely more on environmental resources than on complex interspecies interactions. From the viewpoint of functional redundancy, this can also influence the structural characteristics of bacterial communities (Allison and Martiny, 2008; Miki et al., 2013; Louca et al., 2018). Higher bacterial diversity in SW may lead to increased functional redundancy, wherein different species perform overlapping metabolic functions. Such redundancy can enhance community stability by reducing competitive pressure and promoting species coexistence. However, it may also weaken interspecific functional dependencies, thereby diminishing co-occurrence strength and simplifying the symbiotic network structure.

### Differences of bacterial community mechanisms in different media

Understanding the ecological assembly mechanisms of bacterial communities across distinct environmental media is essential for microbial ecology (Morrison-Whittle and Goddard, 2015). However, the processes governing bacterial community construction in ice and water environments, particularly under the influence of cryoconcentration, have not been explored sufficiently. Our results demonstrated that stochastic processes exerted a greater impact than deterministic processes in shaping the bacterial community structure in both BI and SW samples (Figures 7a, b). This pattern is primarily driven by environmental heterogeneity and the dynamic nature of lake habitats, which reduce the strength of environmental filtering, thereby elevating the role of stochastic forces. In ice-covered systems, the ice layers limit the input of dissolved and particulate nutrients from atmospheric and terrestrial sources, further weakening deterministic environmental selection in the water column (Zhou et al., 2024). However, deterministic processes remain ecologically relevant. This study observed that extreme environmental conditions, such as low temperature and nutrient scarcity, enhanced environmental filtering within the ice layer and increased the relative contribution of deterministic mechanisms in BI community assembly (Swenson and Enquist, 2009). Consequently, the contribution of deterministic processes in SW was reduced compared to BI. This pattern is consistent with the bacterial community assembly mechanisms observed in lake water during ice-free periods. During ice-free periods, nutrient migration within the lake diminishes, resulting in lower nutrient concentrations in the water column than during ice-covered periods. As a result, the associated environmental constraints weaken the assembly of bacterial communities, further reducing the role of deterministic processes in shaping bacterial communities in the water column (Shi et al., 2024). Overall, these findings highlight the influence of cryoconcentration on bacterial community assembly processes.

Specifically, under the combined influence of environmental fluctuations and bacterial ecological traits, bacterial community assembly in ice-covered and water habitats was governed by five ecological processes. Among them, DL and DR, serving as two stochastic processes, were dominant, primarily shaped by low temperatures, nutrient scarcity, environmental heterogeneity, and biotic interactions. HoS accounted for 14% and 7% of community assembly in the BI and SW samples, respectively, whereas HeS contributed only 2% in BI samples. The formation of an ice layer acts as a physical barrier to bacterial dispersal, whereas low winter temperatures suppress metabolic activity and motility, thereby limiting bacterial diffusion (Jiang and Shen, 2007; Newton et al., 2011; He et al., 2021). Previous studies have shown that environmental selection and DL are often interdependent (Lv et al., 2022; Niu et al., 2023). Even when microorganisms possess traits suited to specific environments, they may fail to establish communities if their dispersal is restricted. Conversely, if dispersal is not limiting but environmental conditions are unfavorable, colonization remains unsuccessful. These dynamics help explain why DL could emerge as the primary ecological process driving bacterial community assembly under ice-covered conditions.

Lake environment dynamics play a critical role in regulating the balance between deterministic and stochastic processes during bacterial community succession (Nyirabuhoro et al., 2021). During the freeze-up period, the cryoconcentration effect can facilitate the downward migration and precipitation of nutrients from the ice, increasing the environmental variability within the ice-water interface and shifting the bacterial assembly process toward a new equilibrium state (Manna et al., 2019; Dini-Andreote et al., 2015; Figures 7d, f). In water columns, the availability of nutrient salts remains the principal limiting factor that directly influences bacterial growth and metabolic activity (Manna et al., 2019; Figure 7e). Additionally, changes in ionic strength and osmotic pressure further modulate the community composition by altering cellular homeostasis and microbial tolerance thresholds. When such key limiting factors surpass critical thresholds, the bacterial community may re-establish structural equilibrium, as supported by earlier studies (Yang et al., 2020). In summary, our findings provide direct empirical evidence that cryoconcentration can have divergent effects on bacterial community stability under ice-covered conditions, shaping microbial assembly trajectories through both nutrient redistribution and environmental filtering mechanisms.

## Conclusion

Based on high-throughput sequencing data, this study systematically examined the stability and assembly mechanisms of bacterial communities in Lake Hulun during the ice-covered period, with a particular focus on the effects of cryoconcentration. These results demonstrated that the cryoconcentration effect drove nutrient and microbial redistribution across the ice-water interface, thereby influencing the bacterial community structure. Bacterial diversity and abundance significantly increased from BI to SW, emphasizing the role of nutrient enrichment in shaping the microbial dynamics in ice-covered lake ecosystems. Redundancy analysis identified salinity, TP, and TN as key environmental drivers of bacterial community variation. Despite the predominantly synergistic interactions observed in both BI and SW, the ecological networks in BI exhibited higher connectivity, suggesting that oligotrophic conditions may enhance inter-species exchange. Furthermore, stochastic processes, particularly DL and DR, were the primary forces shaping community assembly, particularly under the constrained environmental conditions imposed by ice cover. The redistribution of TN and salinity under ice-covered conditions played a critical role in stabilizing the bacterial community structure. Overall, this study provides novel insights into microbial dynamics in cold arid lake systems and offers a scientific foundation for future strategies aimed at the conservation and restoration of ice-covered aquatic ecosystems.

## Data Availability

The original contributions presented in the study are publicly available. This data can be found here: https://www.ncbi.nlm.nih.gov/, PRJNA1290662.
